# Novel missense mutations of the *Deleted-in-AZoospermia-Like (DAZL) *gene in infertile women and men

**DOI:** 10.1186/1477-7827-4-40

**Published:** 2006-08-02

**Authors:** Joyce Y Tung, Mitchell P Rosen, Lawrence M Nelson, Paul J Turek, John S Witte, Daniel W Cramer, Marcelle I Cedars, Renee A Reijo-Pera

**Affiliations:** 1 Human Embryonic Stem Cell Center; Center for Reproductive Sciences; Department of Obstetrics, Gynecology, and Reproductive Sciences; Department of Urology; University of California at San Francisco, San Francisco, CA 94143, USA; 2 Developmental Endocrinology Branch; Intramural Research Program, National Institute of Child Health and Human Development; National Institutes of Health, Bethesda, MD 20892, USA; 3 Department of Epidemiology and Biostatistics; University of California, San Francisco, San Francisco, CA 94143, USA; 4 Epidemiology Center; Brigham and Women's Hospital, Boston, MA 02115, USA

## Abstract

**Background:**

The *Deleted-in-AZoospermia-Like (DAZL) *gene has homologs required for germ cell development in many organisms. Recently, we showed that there are several common polymorphisms within the *DAZL *gene that are associated with age at ovarian failure/menopause and sperm count.

**Methods:**

Here we sought to identify rare mutations in *DAZL *and examine their phenotypes in men and women. We sequenced the *DAZL *gene in 519 individuals; sequences spanned the entire coding region of the gene.

**Results:**

We report the identification of four putative missense mutations in *DAZL*. Three individuals that were heterozygous for a *DAZL *mutation reported having children, while two individuals that were homozygous reported no children. These mutations were found only in infertile men and women.

**Conclusion:**

Given the strong data associating *DAZL *polymorphisms and deletions with fertility in humans and model organisms, we suggest that these mutations may be associated with age at menopause and/or sperm count and warrant further biochemical and genetic investigation.

## Background

Though infertility affects 10–15% of couples, only a small number of genes have been shown to be associated with infertility in women and men [1]. In women, family history is a significant predictor of early menopause (menopause at age < 47 years); a woman with an immediate family member who experienced an early menopause has a 6-fold greater risk of early menopause herself [2,3]. In addition, sibling studies have estimated the heritability of age at onset of menopause to be high, 63% in one study and 85% in another [4,5]. In men, there are fewer studies that have examined the genetics of sperm production; most have focused on the role of the Y chromosome [6-8]. Indeed, the most common genetic lesion appears to be deletions of the Y chromosome, including deletions of the *DAZ *(*Deleted-in-AZoospermia*) gene, which are associated with azoospermia (no sperm in the ejaculate) and oligozoospermia (< 20 million sperm/mL of ejaculate) [6-8].

As many features of germ cell development are similar in both sexes, we expect that some genes may function similarly in men and women. In humans and other mammals, male and female germ cells initially develop along the same path, but take different courses after the mitotic expansion of premeiotic germ cells [9]. Oocytes are lost through atresia (apoptosis) and ovulation. With no oogonial stem cell population, eventually the oocyte population is depleted, triggering menopause [10]. Men, on the other hand, have a reserve of spermatogonial stem cells that remains throughout life, and can produce mature gametes continually [9]. One likely candidate gene that may be required for fertility in both men and women is the *DAZL *(*DAZ-Like*) gene, an autosomal homolog of the Y chromosome *DAZ *gene [8,11]. In all organisms studied thus far, *DAZL *expression is specific to germ cells, and mutations in *DAZL *homologs are limited to defects in development of the germ cell lineage [11-27]. We thus hypothesized that mutations in *DAZL *could impact measures of germ cell numbers in men and women, namely sperm count and age at menopause, respectively.

In a previous study, we directly sequenced the human *DAZL *gene in three study populations [28]. The first population was comprised of 93 women diagnosed with idiopathic spontaneous premature ovarian failure (premature ovarian failure group). The second population was comprised of a case control group that contained 324 women with reported ages of ovarian failure/menopause between 28 to 54 years (ovarian failure/menopause group) [2]. The third population contained 102 infertile men with few or no sperm (oligozoospermia, < 20 million sperm/ml), and/or immotile sperm (asthenozoospermia, < 50% motile sperm) (infertile male group). In these three groups, we identified 95 sequence variants within the eleven exons and flanking regions of *DAZL *[28]. A comparison of all the variants revealed twelve SNPs (single nucleotide polymorphisms) or variants that were in Hardy-Weinberg equilibrium and had frequencies, of the least common allele, of greater than one percent. Of the twelve common SNPs, one resulted in a non-synonymous amino acid substitution, six altered nucleotides of the 3'UTR, and five mapped to introns [28]. Our work further demonstrated a strong association between several SNPs and age at menopause in women and sperm count in men, across ethnicities, suggesting that *DAZL*, as in other organisms, may have a function in human germ cell development [28]. Notably, that study was focused on identifying and characterizing common variants in men and women. Here we examined variants that were of low prevalence in the population and thus would constitute mutations based on their frequency. Below, the identity of these putative mutations, and their associated phenotypes, are described in more detail.

## Methods

### Study populations

Study populations and DNA samples are as previously described [28]. Briefly, DNA samples from the premature ovarian failure group were collected from women with premature ovarian failure (age range: 13–41 years). Age at menopause was defined as amenorrhea for at least four months, with two serum FSH (follicle stimulating hormone) levels in the menopausal range one month apart [29]. Causes of ovarian failure such as X chromosome translocations and deletions were excluded. DNA samples from the ovarian failure/menopause group were collected from women with ovarian failure/early menopause prior to or after the age of 46, as described [2]. One third of the women in this population reported an early menopause and a family history of early menopause (prior to age 46, N = 108), one third reported an early menopause and no family history (N = 108), and the remaining third reported menopause after the age of 46 or were still menstruating at age 46, and served as controls (N = 108). Samples were obtained only from the women surveyed and not from their family members. DNA samples from the infertile male group were collected from infertile men; each man in this group had a complete history, physical exam, karyotype, semen analysis performed according to WHO criteria, and testicular biopsy when appropriate [30]. In the case of multiple semen analyses with different reported values, the semen sample with the highest sperm count was included in statistical models.

### DNA extraction and genotyping

DNA extraction and genotype were performed as described [28]. The entire coding region of *DAZL *was sequenced, including all exons and flanking regions. Primers, primer concentrations, and PCR conditions were as indicated [28]. PCR primers were designed using Primer3, with or without the human repeat mispriming library [31]. Sequencher v4.1.4 (Gene Codes, Ann Arbor, MI), Seqman (DNAStar Inc., Madison, WI) and Mutation Surveyor v2.1 (SoftGenetics, State College, PA) were used to align sequences and to identify polymorphic bases.

## Results and discussion

**Figure 1:**
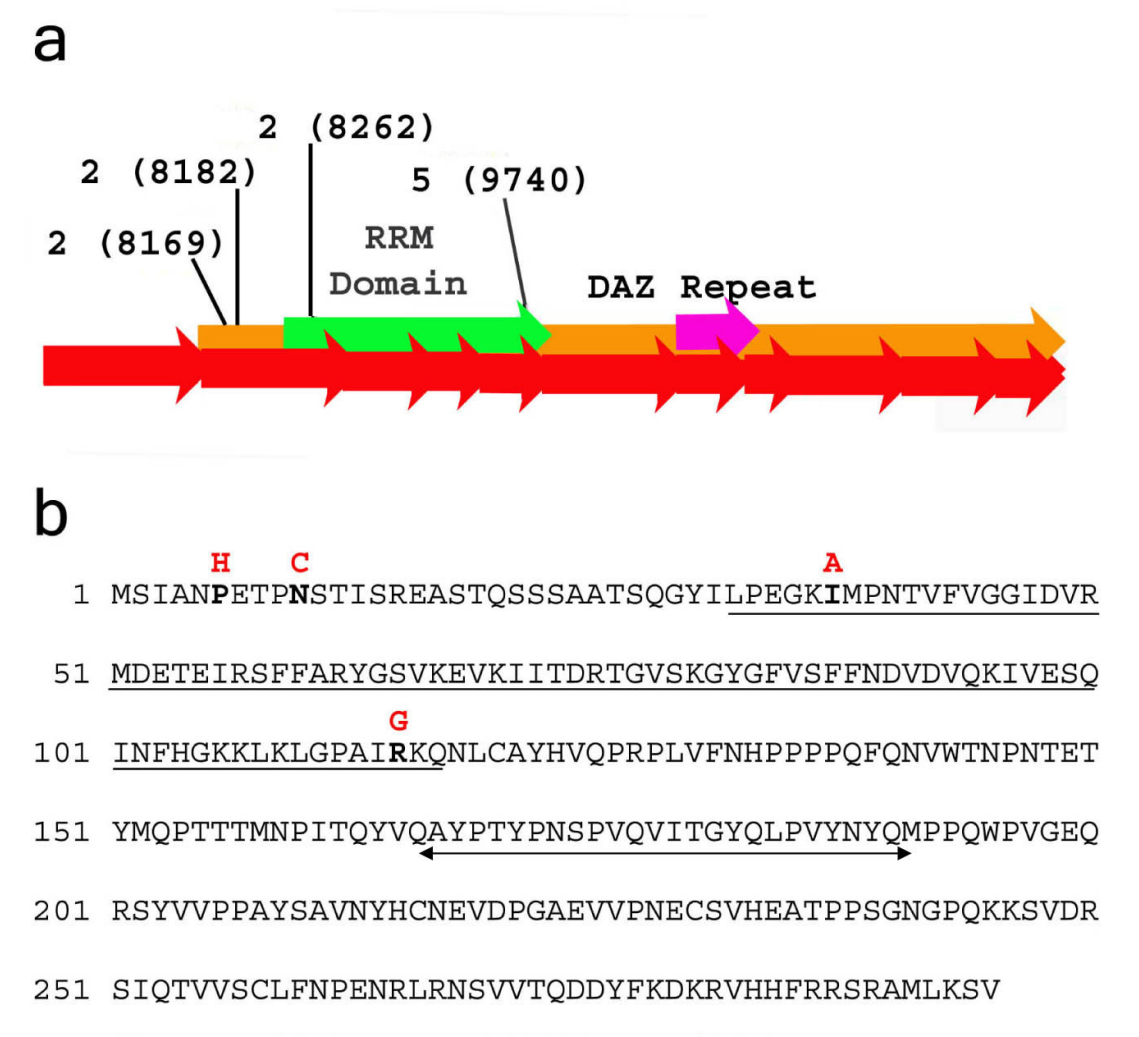
**Four putative missense mutations in the DAZL protein**. (a) Pictoral representation of the *DAZL *gene, with exons depicted by red arrows and the coding region depicted by an orange arrow. Two protein motifs, the RNA Recognition Motif (RRM) and the DAZ repeat, are represented by green and pink arrows, respectively. Each mutation is labeled by its exon and its position in the gene, which is mapped to the sequence as previously reported [28]. (b) Sequence of the DAZL protein, with mutated residues in bold, and the resulting changed amino acid above in red. The highly conserved RRM, which is required for RNA binding, is underlined (LPE........to IRK) [36, 42]. Another highly conserved domain of the protein, the DAZ motif, is underlined with double arrows (AYP....... to NYQ).

Single nucleotide polymorphisms (SNPs) are defined as naturally-occurring variants with a frequency greater than one percent in the human population; in contrast, mutations occur with frequencies less than one percent. Here, we identified four putative missense mutations in the *DAZL *gene, listed in Table 1 and depicted in Figure 1, that were found only in one or two individuals in all three populations that we studied. None of these mutations were identified in the normal controls (from the ovarian failure/menopause group). Three mutations mapped to exon 2 and one mapped to exon 5 of the *DAZL *gene; all were juxtaposed to, or within, the RNA-binding domain.

**Table 1:** Missense mutations identified in the human *DAZL *gene. The position of each putative mutation in the *DAZL *gene sequence is relative to the gene sequence as previously detailed [28].

**Position**	**Exon**	**Change**	**Occurrence**		
			
			**Sex (Population)**	**Genotype**	**Phenotype**
8169	2	Pro6 → His6	Female (ovarian failure/menopause group)	Heterozygous	Spontaneous early menopause at age 45. 3 children previously.
8182	2	Asn10 → Cys10	Male (infertile male group)	Homozygous	Azoospermia
			Female (ovarian failure/menopause group)	Heterozygous	Familial early menopause at age 44. 4 children previously.
8262	2	Ile37 → Ala37	Female (ovarian failure/menopause group)	Heterozygous	Spontaneous early menopause at age 43. 1 child previously.
9740	5	Arg115 → Gly115	Female (premature ovarian failure group)	Homozygous	Spontaneous premature ovarian failure at age 34. 0 children previously.

### Pro6 → His6

This mutation changes a highly conserved proline to a histidine in the N-terminus of the protein, just upstream of the highly-conserved RNA Recognition Motif (RRM). Although proline is usually considered a hydrophobic amino acid whose unique cyclic structure causes it to influence protein architecture, its secondary amino (NH_2_) group predisposes this residue to being on the surface of the protein exposed to water. Histidine is also a bulky amino acid that can be either neutrally or positively charged, depending on its environment. Overall, since both amino acids are large and generally localized to the surface of the protein, this amino acid change is most likely to affect the structure of the protein surface. In this study, we identified one woman who was heterozygous for this mutation, with onset of menopause at age 45, and no family history of early menopause. She was fertile, having had three children prior to menopause.

### Asn10 → Cys10

This amino acid change was identified in two patients: a male homozygous for the mutation who presented with azoospermia and a female heterozygous for the mutation who reported an early menopause at age 44. Notably, asparagine is a negatively charged hydrophilic residue, while cysteine contains the hydrophobic and highly reactive sulfhydryl group. This particular change lies in exon 2 just outside of the RNA-binding domain; the substitution of this hydrophilic residue for a hydrophobic residue may impact protein folding. Interestingly, in this case, the mutation may act in a dose-dependent manner: the woman with one copy of the mutation had a less severe phenotype than the male with two copies of the mutation.

### Ile37 → Ala37

This isoleucine is part of the RNA-binding domain (the RRM or RNA Recognition Motif). It is five amino acids upstream of the RNP2 sequence which is one of the most highly conserved parts of the DAZL protein. Isoleucine and alanine are both classified as hydrophobic amino acids; however, alanine, with its shorter side chain, is not as hydrophobic as isoleucine. The structure of the homologous RNA binding domain in another protein has been deduced, and in that structure, the amino acid corresponding to the isoleucine forms a hydrogen bond with another amino acid in the structure [32]. The shorter side chain of alanine would be expected to disrupt this hydrogen bond. This mutation was only identified in a woman who experienced a spontaneous early age at onset of menopause at age 43. She was heterozygous for the mutation and had one child.

### Arg115 → Gly115

Arg115 is near the carboxy-terminal end of the RRM in a region that interacts with several other proteins [33]. This mutation results in the substitution of a basic, hydrophilic amino acid for the simplest amino acid, glycine. By sequence comparison to the RRM of another RNA-binding protein, the HuD protein which has been crystallized bound to RNA, it appears that this amino acid may be key to making side-chain and main-chain connections to the RNA and the other RRM in this protein [34]. As DAZL is also known to homodimerize, this amino acid may be important for both RNA binding and protein-protein interactions. Substitution of arginine by glycine should severely disrupt normal interactions with this amino acid. Accordingly, the one patient we identified with this mutation was homozygous for this change, and experienced premature ovarian failure at age 34, having borne no children.

### *DAZL *mutations and reproductive characteristics

Our screen of the *DAZL *gene for sequence variations that are associated with male and female fertility produced not only a number of interesting common SNPs or variants [28], but also intriguing rare missense mutations, as reported here. None of these mutations were found in the 108 control women that had a normal menopause or were still menstruating at age 46, suggesting they may be associated with impaired germ cell development and fertility. Nonetheless, additional data is required; for example, it would be most useful to independently screen a second group of male and female control chromosomes to determine whether these mutations are found in individuals with "normal" fertility/reproductive characteristics. This is especially merited given that these mutations were clustered in exons that form part of the RRM. While it is possible that this region of the protein is simply highly variable, it is more likely that our population is enriched for mutations in a functionally-important part of the protein. In other RRM-containing proteins, many of the residues surrounding the RRM motif are also involved in RNA binding specificity and thus, even those mutations that are adjacent to the RRM may alter proper protein function [35].

Interestingly, we observed that possessing these putative missense mutations, especially a heterozygous missense mutation, was compatible with producing offspring. Each patient that was heterozygous for a missense mutation was able to produce at least one child. In contrast, the patient that was homozygous for the Arg115Gly mutation experienced 46,XX spontaneous premature ovarian failure at the early age of 34 years and did not bear any children; similarly, the male patient that was homozygous for the Asn10Cys mutation produced no sperm. This is consistent with data from the mouse, where mice heterozygous for a *Dazl *knockout allele are fertile (though it is currently debated whether they may be subfertile) and homozygous mutant mice are infertile in both sexes [17].

Recently, a number of potential RNA targets for DAZL have been identified, making it possible to test the effects of mutations on RNA-binding *in vitro*, in the future [36-39]. In addition, as this region is known to be involved in DAZL homodimerization and binding to other proteins, it would be of interest to determine whether these mutations, like Arg115Gly for example, affect the ability of DAZL to interact with other protein partners [33,40,41].

## Conclusion

Intriguing mutations were observed in the *DAZL *gene in both men and women. Two individuals possessed homozygous mutations. Future work on how these putative mutations affect RNA and protein partner binding could provide key insights into the requirement of *DAZL *in human germ cell development, especially regarding key residues required for DAZL protein function. In addition, further studies may shed a light on the role of *DAZL *in human infertility.

## Competing interests

The author(s) declare that they have no competing interests.

## Authors' contributions

JT and MR carried out the molecular genetic studies, sequence alignment and drafted the manuscript. LMN, PJT, DWC, and MIC participated in the design of the study, especially clinical aspects. JSW performed statistical analysis. RARP conceived of the study, and participated in its design and coordination and helped to draft the manuscript. All authors edited, read and approved the final manuscript.
